# Chinese Herbal Medicines in the Treatment of IBD and Colorectal Cancer: A Review

**DOI:** 10.1007/s11864-014-0288-2

**Published:** 2014-05-04

**Authors:** Maciej Sałaga, Hubert Zatorski, Marta Sobczak, Chunqiu Chen, Jakub Fichna

**Affiliations:** 1 Department of Biochemistry, Faculty of Medicine, Medical University of Lodz, Mazowiecka 6/8, 92-215 Lodz, Poland; 2 People’s Hospital of Shanghai, School of Medicine, Tongji University, Shanghai, China

**Keywords:** Traditional Chinese medicine, Herbal medicine, Inflammatory bowel diseases, Colorectal cancer

## Abstract

Inflammatory bowel diseases (IBD) are a group of chronic inflammatory gastrointestinal (GI) disorders, mainly represented by Crohn's disease and ulcerative colitis. Although the etiology of IBD is not fully understood, there is substantial evidence that immunologic, genetic, and environmental factors are the main contributors in IBD pathogenesis. Conventional therapies for IBD include anti-inflammatory and immunosuppressive drugs, such as 5-aminosalicylic acid, corticosteroids, antibiotics, and biologicals, such as anti-TNFα antibodies. However, because of low efficacy and high risk of side effects, there is a clear need for the development of novel and efficient pharmacologic strategies in IBD treatment. Among various complementary and alternative medicine (CAM) approaches, which are used for the treatment of gastrointestinal (GI) disorders, traditional Chinese medicine (TCM) is one of the most developed and diversified. TCM encompasses methods and therapies that emerged over centuries and is based mostly on ethnic wisdom and observations transmitted from generation to generation. In the recent years, the efficacy of TCM as treatment of IBD has been extensively characterized in preclinical and clinical studies, which resulted in a significant number of research reports. Moreover, the popularity of TCM among patients with IBD has rapidly increased not only in Asia, but also in the Western hemisphere.

## Introduction

Inflammatory bowel diseases (IBD) is a group of chronic inflammatory gastrointestinal (GI) disorders, consisting of Crohn’s disease (CD) and ulcerative colitis (UC) that may cause a substantial worsening of life quality. Patients suffering from IBD often require lifelong pharmacologic treatment and sometimes even surgical interventions. To achieve a successful management of IBD, strong adherence to medication is essential and addressing patients’ physical and perceptual barriers to adherence is crucial in improving their health status [1••]. Nowadays, conventional medicine is offering a broad range of anti-IBD drugs, including corticosteroids, immunosuppressants, antibiotics, and biologicals, such as anti-tumor necrosis factor α (anti-TNF-α) antibodies [2, 3]. Some of these conventional anti-IBD drugs are associated with the risks of adverse events, such as metabolic deregulation leading to obesity (for corticosteroids), or adverse skin reactions for anti-TNF-α antibodies [4, 5]. Moreover, none of the drugs presently available, which are used for anti-IBD therapy, are effective enough to provide complete and life-long relief for the patients.

One of the most threatening risks, which IBD patients are exposed to, is the risk of development of colorectal cancer (CRC) as a result of the chronic inflammatory state in the lower GI tract. Literature data indicates that chronic inflammation, which underlies colitis-associated colorectal cancer (CACRC), may contribute to the development of up to 25 % of all diagnosed colorectal tumors [6]. Chronic accumulation of active neutrophils, macrophages, and dendritic cells in the lamina propria and the colonic mucosa during inflammation is accompanied by the release of several cytokines, such as TNF-α and IL-1β [7], as well as reactive nitrogen and oxygen species [8], which are known to induce genetic mutations. Subsequently, the DNA damage and instability of microsatellite sequences in the colonic cells may result in activation of proto-oncogenes. For example, critical roles of activated NF-κB, phospho-NF-κB and STAT3, which act as nonclassical oncogenes in CRC and other cancers have been reported [9]. The most effective methods of CACRC treatment are cytotoxic drugs, radiotherapy, or resection of tumor area. Low selectivity between cancer and healthy cells and a major risk of side effects, often combined with poor efficacy and the risk of relapse are the main reasons for which new therapies against CRC are urgently needed [10].

Over the last few years the popularity of complementary and alternative medicine (CAM) among IBD and CACRC patients has been systematically increasing, mainly because it is perceived as natural and effective [1••]. CAM generally denotes medical theories and practices, which are outside the realm of conventional, western medicine. Another feature, which separates CAM from conventional medicine, is a holistic approach to patient care, calling on self-healing by the body and being applied in an individualized way [11]. CAM encompasses a vast and strongly diversified spectrum of therapeutic procedures, as well as systematic and comprehensive concepts of disease treatment, which includes traditional practices arising from tribal traditions and ethnic wisdom. Practices of alternative medicine are often based on ideas, which discriminate modern pathophysiology and pharmacology, relying more on traditional practices and on ‘natural’ remedies, which are being perceived as less toxic than conventional drugs [11]. One of the common features of CAM is a minor number of scientific data available in the form of controlled trials for either its efficacy or safety.

Various studies, including cohort and population-based surveys, have shown that the use of CAM, with acupuncture, Ayurvedic medicine, homeopathy, and herbal medicine, is common among IBD patients from all age groups [1••, 11–15]. The percentage of IBD patients using CAM in North America and Europe has been estimated at 21 % up to even 60 % [1••]. In a recently published systematic review on herbal therapy in IBD by Ng et al [1••], it has been shown that younger age, female gender, higher education level, adverse drug reactions from IBD medication, extra-intestinal manifestations, perceived stress, and prolonged and intensive courses of steroids are associated with the preference to use of CAM by IBD patients over other forms of therapy [1••].

Traditional Chinese medicine (TCM) is one of the most developed branches of CAM in the world. Methods offered by TCM range from acupuncture, through homeopathy, to the vast number of herbal preparations, often of yet unknown composition and mechanism of action. In this review we refer to the use of TCM in the treatment of GI tract disorders, with particular attention given to IBD and CACRC. We will try to shed a light on the effectiveness of different practices and therapeutic preparations derived from TCM. We will also discuss experimental in vitro and in vivo results obtained from systematic literature search, carried out by consulting 13 electronic scientific databases including MEDLINE, SCOPUS, Web of Science, Directory of Open Access Journal; electronic editorial networks, such as BMJ, Blackwell, Elsevier, Karger, Nature Publishing Group, Springer; literature distributors, such as OVID Journals and EBSCO; clinical trials databases, such as CT and EU - CTR. The scientific papers were selected according to the time span ranging from 2001 to present in case of in vivo studies on animal models and from 1997 in case of human studies because of the lesser amount of the latter in the literature.

## Treatment

### Inflammatory bowel diseases

- Depending on the extent of disease, management of IBD requires specific diet, pharmacologic treatment, and/or—in some cases—surgery. First line of classical pharmacologic therapy includes nonsteroid and steroid anti-inflammatory drugs (eg, mesalazine, budesonide), which is often time-limited because of potential adverse events. The second treatment option includes biologicals, such as anti-TNFα antibodies (eg, infliximab). In severe cases of IBD a surgery is required, which involves resection of the fragment of the inflamed intestine. CAM methods are used in the group of patients where classical treatment did not provide sufficient relief. Several scientific reports characterizing the effectiveness of TCM products in animal models of IBD, as well as clinical studies have been published so far, showing promising results and providing an insight into the mechanisms that are beyond their therapeutic activity (Table 1). Here, selected TCM medications, which recently attracted most attention, will be discussed.

**Table 1:** Summary of in vivo studies involving TCM compounds used in anti-IBD and anti-CRC therapy

TCM drug	Model	Comparator	Route of administration	Observation	Potential mechanism of action (comments)	Ref.
YunNan BaiYao	DSS- and TNBS-induced colitis	5-ASA, 6-MP	in drinking water (p.o.)	Improvement of colitis/abolished inflammation	Immune-suppressing (reduction of TNFα, IL-12, -17), wound-healing	[16]
W.chinensis water extract	DSS-induced colitis	No data	p.o.	Improvement of colitis/abolished inflammation	Immune-suppressing (suppression of Th1 and Th17 cells)	[17]
Changtai granules	Chronic TNBS-induced colitis	DX	i.c.	Improvement of colitis/abolished inflammation	Immune-suppressing (suppression of macrophages/monocytes)	[18]
G. frondosa water extract	TNBS-induced colitis	5-ASA	p.o.	Reversed symptoms of colitis	Immune-suppressing (reduction of TNFα, NF-κB and neutrophil influx)	[21]
Changlu Enema	Protein immunization induced-UC	SASP	i.c.	Reversed inflammatory response (macro- and microscopically)	No data	[22]
Mixed herbal preparation	DNCB and acetic acid-induced colitis	SASP	i.c.	Improvement of colitis/abolished inflammation	Immune-suppressing (reduction of TNFα, INFγ and IL-6)	[23]
β,β-demethylacrylshikonin	HCT-116 tumor xenograft	None	i.p.	Reduction of tumor growth	inducing apoptosis of tumor cells (increasing level of Bax/ decreasing level of Bcl-2)	[41]
Shikunshito-Kamiho	DMH-induced CRC	None	in diet (p.o.)	Reduction of aberrant crypt foci	No data	[42]
Sophoridine	SW480 tumor xenograft	5-FU	i.p.	Reduction of tumor growth	inducing apoptosis of tumor cells ( activation of caspase-3 dependent pathways)	[45]
Spica Prunellae ethanol extract	HT-29 tumor xenograft	None	p.o	Reduction of tumor growth	induction apoptosis of tumor cells/inhibition of tumor angiogenesis and proliferation	[46]

*5-ASA* 5-aminosalicylic acid, *5-FU* 5-fluorouracil, *6-MP* 6-mercaptopurine, *CRC* colorectal cancer, *DMH* 1,2-dimethylhydrazine, *DNCB* 2,4-dinitrochlorobenzene, *DS* dextran sodium sulfate, *DX* dexamethasone, i.c. intracolonic, *i.p.* intraperitoneal, *p.o.* per os, *SASP* salicylazosulfapyridine, *TNBS* 2,4,6-trinitrobenzenesulfonic acid, *UC* ulcerative colitis

### Chinese traditional medicine and IBD (animal studies)

- In 2011, Li et al [16] characterized the anti-inflammatory activity of YunNan BaiYao (YNBY), aforetime also called Yunnan Pai-yao, which is an amalgamation of various herbs and plants derived from southern China formulated in powder or capsule [16]. In China it is used as a low-cost medication for both, internal and external hemorrhage and open wounds, mainly because of its well-known anti-hemorrhagic and hemostatic functions. Besides the paper by Li et al there is a lack of English language literature concerning preclinical or clinical evaluation of YNBY activity. Interestingly, the exact composition of YNBY is not widely known, because it is secured as a Chinese intellectual property by the government [16]. Li et al reported that YNBY can effectively reduce the severity of experimental colitis in mice by both, immune-suppressing and wound-healing mechanisms. It was shown that the administration of YNBY in drinking water significantly reduced disease progress in DSS- and TNBS-induced colitis. In both models YNBY reduced the levels of pro-inflammatory cytokines, such as TNFα, IL-12, IFNγ, and IL-17 in colon and serum [16]. Moreover, YNBY suppressed the growth of T- and B-lymphocytes more significantly than the most commonly used first-line anti-IBD drugs, 6-mercaptopurine (6-MP) and 5-aminosalicylic acid (5-ASA), showing no cytotoxicity to colonic cells (Caco-2).
- In another study Huang et al [17] reported the anti-inflammatory activity of 4 different *Wedelia chinensis* extracts in a mouse model experimental colitis induced by DSS; water extract exhibited the highest anti-inflammatory efficacy. *W. chinensis* is a traditional medicinal herb, widely used in many Asian countries, with various therapeutic properties, including cough-relieving, antipyretic, anti-inflammatory and also hepato-protective [17]. Huang et al observed that *W. chinensis* water extract significantly attenuated the symptoms of colitis including diarrhea, rectal bleeding, and loss of body weight [17]. It also reduced the shortening of colon length and histopathologic damage caused by colonic inflammation [17]. The anti-inflammatory activity of *W. chinensis* water extract possibly results from its immunosuppressive activity on T-cells. It has been demonstrated that oral administration of *W. chinensis* affected the levels of regulatory cytokines (TNF-α, IL-4, IFN-γ, IL-17, TGF-β, and IL-12) showing that this kind of treatment can suppress Th1 and Th17, but not Th2 responses in colon tissues of DSS-treated colitic mice [17]. Moreover, *W. chinensis* water extract proved to be nontoxic to mice when administrated at high doses (100–1000 mg/kg) [17].
- Cao et al reported the effect of Changtai granules (CTG) on chronic TNBS-induced colitis in rats [18]. CTG is a traditional Chinese formula consisting of herbs, such as Huangbai (*Phellodendron amurense*) [18]. CTG exhibits a variety of pharmacologic effects including analgesic, anti-bacterial, anti-inflammatory and anti-diarrheal [18]. The study of Cao showed that CTG was more potent than dexamethasone (DX) in amelioration of colitis in rats, but it should be noted that CTG was used in a substantially higher dose than DX. CTG effectively inhibited the activity of granulocytes, macrophages, and monocytes in a dose-dependent manner. It also caused a substantial decrease in myeloperoxidase (MPO) activity and ulceration in colonic mucosal tissue, thus the immunosuppressive mechanism of CTG action has been suggested. Furthermore, administration of CTG significantly prevented body mass loss, and decreased frequency of diarrhea in TNBS-treated rats, when compared with the control animals [18].
- For thousands of years, mushrooms have been considered in TCM as an edible medicinal resource. For example, *Grifola frondosa* (GF) has been used as a remedy for pain and inflammation in Southeast Asia. Studies demonstrated that extracts from fruiting body or liquid-cultured mycelium of GF display considerable biological activities, such as anti-tumor, anti-mutagenic, anti-hypertensive, anti-diabetic and antioxidative [19–21]. Lee et al have shown that oral administration of GF water extract (GFW) at the dose of 1 g/kg reversed the symptoms of colitis (weight loss, colon ulceration, TNF-α expression in the tissue) to the same extent as 5-ASA at the dose of 0.1 g/kg (i.p.) [21]. Moreover, GFW significantly reduced the MPO activity, indicating the reduction of neutrophil influx into the colonic tissue. Additionally, Lee et al tested GFW in the in vitro model of IBD on HT-29 cells. They demonstrated that in the coculture of HT-29 with U937 human monocytic cells, the TNF-α-induced monocyte adhesion to HT-29 cells was significantly suppressed by GFW (10, 50, and 100 μg/ml) [21]. The GFW-induced reduction of adhesion was correlated with the suppressed expression of MCP-1 and IL-8, major IBD-associated cytokines and decreased transcriptional activity of NF-κB [21]. These results suggest that GFW alleviates colonic inflammation by suppressing production of TNF-α and its signaling through NF-κB, which results in a lowered expression of pro-inflammatory chemokines [21].
- TCM medications for GI disorders are often in the form of herbal retention enema. Recently, Wu et al evaluated the therapeutic effect of Changlu Enema (CE) in the mouse model of UC, induced by colonic mucosa protein immunization [22]. Authors showed that after 21 days of medication the UC activity index was significantly lower and the body weight was higher than in the mice treated with saline only. Moreover, it has been shown that pathologic (microscopic) changes and inflammatory response in colonic tissue were reversed after treatment with CE and the improvement was more significant than in the salicylazosulfapyridine (SASP)-treated group [22]. In another study Guo et al characterized the pharmacologic action of a TCM herbal enema in the rat model of UC induced by administration of 2,4-dinitrochlorobenzene (DNCB) and acetic acid [23]. The formulae of TCM herbs for enema consisted of Huangqi (*Astragalus*), Dahuang (*Caulis Fibraureae*), Huangbai (*Cortex Phellodendri*), Wubeizi (*Galla chinensis*) and Baiji (*Rhizoma Bletillae*) [23]. The study showed that the TCM herbal enema was more potent than SASP in the treatment of UC [23]. Moreover, it has been showed that treatment with TCM herbal enema resulted in a reduced expression of IL-6, TNF-α and IFN-γ [23]. The authors suggested that the mechanism of therapeutic action of TCM herbal enema is associated with the regulation of immune response in the colonic mucosa and inhibition of macrophage/monocyte and granulocyte activity.

### Chinese traditional medicine and IBD (human studies)

- Even though some authors believe that the attempts to characterize the effectiveness of TCM products using randomized-controlled trials (RCTs) are deceptive in view of their exclusion from the realms of scientific hypotheses and difficulties in formulation of endpoints, there are several TCM products that successfully passed animal studies and were introduced to trials on IBD patients (Table 2) [11, 24, 25]. Zhang et al evaluated the anti-inflammatory activity of Xilei-san, a traditional Chinese herbal medicine, in an 8-week double-blinded randomized study on 35 subjects with mild-to-moderately active ulcerative proctitis [26]. The effect of Xilei-san was compared with DX enemas. The study showed that both treatments produced a significant improvement of clinical, endoscopic, and histologic score [1, 26].

**Table 2:** Summary of human studies involving TCM compounds used in anti-IBD and anti-CRC therapy

TCM drug	No. of patients	Comparator	Outcome	Comments	Ref.
Xilei-san	35	DX enema	Improvement of clinical, endoscopic and histologic scores		[26]
Xilwi-san	30	Placebo	Induced remission, lowered relapse rate	Additional treatment: mesalazine, corticosteroids	[27]
Kui jie qing	95	Sulfalazine, prednisolone, prednisone	72 % of cure and 23 % of improvement	Unclear results/conclusions	[28]
HMPL-004	224	Placebo	Greater clinical response than placebo	did not affected 8 weeks remission rate	[29••]
HMPL-004	120	Mesalazine	No significant differences between groups (clinical response, remission rate)	Patients with mild-to-moderate UC	[32]
TWHF	20	None	Rapid reduction of CDAI in first 8 weeks	Prospective study, patients with active CD	[34]
TW	45	Mesalazine	No significant differences between groups (relapse rate)	Patients with postoperatiwe CD	[35]
TW	39	SASP	No significant differences between groups (endoscopic recurrence of disease)	Patients with postoperative CD	[36]
Mixed herbal preparation	78	ETIASA (mesalazine)	Group treated with both oral intake and enema showed best overall clinical effectiveness	No differences between patient treated with enema only and comparator	[37]
Mixed herbal preparation	222	radical operation/chemotherapy/radiotherapy alone	Group treated with oral intake showed lower relapse/metastasis rates	Prospective study	[48]
PHY906	17	chemotherapy alone	Reduction in incidence of diarrhea in treated group	Double-blinded, randomized, placebo controlled, dose escalation, cross-over study	[49]
Quxie Capsule	40	placebo	Reduction in fatality rate, prolongation of survival time, improvement of quality life	Randomized controlled trial	[50]
Quxie Capsule, Fuzheng Capsule	101	None	Higher rate and time of relapse and metastasis in TCM treated group	prospective study	[51]

*CD* CrCD Crohn's disease, *CDAI* Crohn's disease activity index, *DX* dexamethasone, *SASP* salicylazosulfapyridine, *TW* TripterTripterygium wilfordii, *TWHF Tripterygium wilfordii* Hook F, *UC* ulcerative colitis
- In another RCT, Fukunaga et al characterized the potential of Xilei-san to induce remission in 30 patients with intractable ulcerative proctitis [27]. Subjects were treated with topical mesalazine or corticosteroids (4 weeks) and then randomized into Xilei-san suppositories or placebo (2 weeks). In the Xilei-san-treated group, significantly more patients achieved remission (clinical disease index ≤4) compared with placebo (*P* < 0.04) [27]. Histologic improvements, as well as relapse rate at 180 days were significantly lower in Xilei-san-treated group compared with placebo [1••, 27].
- A TCM product called Kui jie qing (KJQ) was used in a form of enema given 4 times daily to 95 patients with active UC [28]. This form of treatment was compared with conventional anti-IBD drugs, including suplhasalazine (1.5 g 3 times daily), oral prednisolone (30 mg once daily) and prednisone enemas (20 mg 4 times daily). After 20 days of treatment authors reported a 95 % effectiveness rate for KJQ and 62 % for conventional drugs, based on the comparison of cure and improvement between the groups [28]. However, results presented in this study cannot be considered conclusive as authors did not provide clear definition of terms “cure” and “improvement”, which were used in this paper as major determinants of final conclusion [1••, 11].
- A recent paper by Sandborn et al demonstrates results from a large randomized double-blinded, placebo controlled trial comparing the extract from an Asian plant *Andrographis paniculata* (HMPL-004) to placebo in 224 patients with UC [29••]. *A. paniculata* is cultivated in Southeastern Asia, where it has been traditionally used to treat infections. For millennia *A. paniculata* was used in traditional, as well as tribal medicine in China, India, and some other countries. In vitro, as well as in vivo studies showed that extracts from *A. paniculata* inhibit formation of oxygen derived free radicals and display anti-inflammatory activity. The major active compound in *A. paniculata* extracts is andrographolide, which blocks the activity of NF-κB, known to be the central transcriptional factor of genes involved in immunologic responses [30, 31]. Sandborn et al showed that treatment with HMPL-004 in a dose of 1.8 g daily resulted in a greater clinical response than placebo (*P* = 0.018), but did not affected the remission rate in 8 weeks (*P* = 0.101) [29••]. A second randomized double-blinded, placebo controlled multicenter study (*n* = 120) showed that an 8-week therapy with HMPL-004 was as potent as mesalazine in the treatment of mild-to-moderate UC [32]. Authors observed no significant difference between experimental groups, thus suggested that HMPL-004 is a promising alternative to mesalazine in UC.
- The anti-inflammatory activity of TCM herbs have also been evaluated in patients with CD. *Tripterygium wilfordii* Hook F (TWHF) is a TCM herbal remedy with immunomodulatory and anti-inflammatory activities. Preclinical studies demonstrated that extracts from TWHF inhibit the expression of pro-inflammatory cytokines, adhesion molecules, and matrix metalloproteinases by macrophages, lymphocytes, synovial fibroblasts, and chondrocytes [33]. Ren et al evaluated the ability of T2, a major constituent of TWHF extracts, to induce the remission in 20 patients with active CD [34]. In this study the patients were treated with T2 pills for 12 weeks (60 mg daily). Crohn's disease activity index (CDAI) scores rapidly decreased during first 8 weeks of treatment and reached minimum in week 10 [34]. Moreover, levels of C-reactive protein (CRP) and pro-inflammatory cytokines, such as TNF-α and IL-1β in plasma significantly decreased at the outset of treatment [34]. It has been also shown that *Tripterygium wilfordii* (TW) may prevent the postoperative recurrence of CD at least to the same extent as mesalazine. In a randomized study by Tao et al 45 adult cases of postoperative CD were randomly divided into 2 groups, which received TW or mesalazine after operation [35]. There was no recurrence of the disease in both groups after 3 months. In 6 months, a similar percent of patients relapsed in TW- and mesalazine-treated groups (18.2 % vs 21.7 %, respectively). In 1 year, 45.5 % of patients in TW-treated group and 60.9 % patients in mesalazine-treated group relapsed. Finally, there were no significant differences between experimental groups during the whole study [35]. In another study, 39 adult postoperative CD patients were enrolled in a randomized, placebo-controlled trial. Patients were treated with TW or SASP in 2 weeks after operation [36]. In the TW-treated group the recurrence of the disease was ascertained in 1 patient (5.6 %) and 22.2 % patients had endoscopic recurrence, compared with 4 patients (25.0 %) and 56.2 % in SASP-treated group [36]. These 2 studies showed that TW is as potent as conventional drugs in preventing recurrence of postoperative CD.
- Recently, Ling et al evaluated the anti-IBD activity of another Chinese herbal preparation administered by oral intake or retention enema in 78 patients for 30 days [37]. The preparation was made of various herbs, including *Radix Astragalus, Radix Codonopsis pilosula, Rhizoma Atractylodes alba, Semen Coicis, Radix Aucklandiae, Radix Angelica Sinensis,* charred *Massa Fermentata Medicinalis, Fructus Hordei Germinatus, Herba Epimedii, Radix Glycyrrhiza, Radix Paeoniae lactiflora alba,* and *Radix Pueraria* [37]. Preparation was administered orally twice daily, at 100 mL each time and/or in the form of the colon retention enema, implemented for 45 minutes every day. The group treated with both, oral intake and enema showed the best outcomes in all aspects of clinical effectiveness and these results were significantly different from other groups [37]. There were no significant differences in main symptoms, colonoscopic scores and histopathology of the colonic membrane between patients receiving TCM preparation by enema only and conventional treatment (ETIASA) [37]. These observations led to the conclusion that TCM herbal drugs administered by both, oral intake and retention enema, are effective in the treatment of IBD. Also, these results indicate that such a mode of therapy, combining both systemic and local administration of TCM herbal drugs is particularly effective in the treatment of IBD.

### Colorectal cancer (CRC)

- CRC is one of the most frequently diagnosed malignant cancers. It is the second most common malignancy in the developed countries and a significant cause of mortality, being the fourth cause of death in the world. The most effective methods of CRC treatment currently available are cytotoxic drugs, radiotherapy or resection of tumor area. In patients with locally advanced rectal cancer, preoperative chemoradiotherapy has proven to significantly improve local control and cause a lower treatment-related toxicity compared with postoperative adjuvant treatment. The low selectivity between cancer and healthy cells and a major risk of side effects, often combined with the risk of relapse are the main reasons for which new therapies against CRC are urgently needed. Of note, the human studies on the efficiency of TCM in CRC cited in this review should be considered with caution because of several weaknesses: lack of important information about blinding, randomizing and excluded or included patients. This review indeed shows some positive findings, but the quality of the trials precludes the making of any definitive statements. Well-conducted randomized clinical trials are needed to confirm any benefit of traditional Chinese herbs in the treatment of CRC.

### Chinese traditional medicine and CRC (animal studies)

- For many years, Shikonin and its derivatives have been used in TCM for the prevention and treatment of various diseases, including cancer. One of the derivatives, β,β-demethylacrylshikonin (DA), a major component of the lithospermum erythrorhizon roots, was identified as a potential inhibitor of hepatocellular carcinoma growth [38, 39]. Further studies reported that DA displays even more pharmacologic properties, such as anti-inflammatory, antioxidant, and antiplatelet [40]. Yingying et al showed that systemic therapy with DA can inhibit tumor growth in animals, which was demonstrated by using HCT-116 xenografts in mice [41]. Once HCT-116 cells were injected subcutaneously into upper left flank region of mice, the animals were divided into 4 groups and treated daily with DA (0.3, 0.6, and 1.2 mg/kg) or saline as a negative control [41]. All mice were sacrificed on Day 13 after inoculation with HCT-116 cells. Treatment with DA significantly reduced tumor growth and exhibited no toxicity, measured by body weight decrease [41]. The mechanism of DA action is arguably associated with inducing apoptosis of tumor cells; the authors found that DA decreased the expression of anti-apoptotic protein Bcl-2 and increased expression of pro-apoptotic Bax protein [41].
- Shikunshito-Kamiho (SKTK) is a TCM preparation composed of 8 crude drugs (*Ginseng radix, Hoelen, Atractylodis Rhizoma, Glycyrrhizae Radix, Prunellae Spica, Ostreae Testa, Laminaria Thallus, Sargassum*), which serves as a cure for CRC in oriental medicine. Yoo et al investigated the anticarcinogenic effect of SKTK on 1,2-dimethylhydrazine (DMH)- induced CRC in mice [42]. The animals received injections of carcinogen (DMH) once a week for 10 weeks while being fed with diet containing 0.5 % or 1.5 % extracts of SKTK [42]. The water extracts of SKTK were provided to the diet for 5 weeks after last injection of DMH. Eventually, the authors evaluated fecal enzymes, such as β-glucuronidase, tryptophase and urease, and the aberrant crypt foci (ACF) formation in murine colon. β-Glucuronidase and tryptophanase were reduced in both groups, whereas the level of urease was significantly reduced only in mice fed with 1.5 % SKTK [42]. The number of ACF in the colon, which are regarded as putative preneoplastic lesions of rodent and humans, was significantly decreased in the group fed with both concentrations of SKTK, compared with positive control (no SKTK) [42].
- Sophoridine (SRI) is one of the quinolizidine alkaloids, which were extracted from seeds of *Saphora alopecuroides* L*.* [43]. This traditional Chinese herb has been used for clearing heat and removing toxins for hundreds of years. Previous research showed that SRI is one of the most active compounds occurring in this herb and can significantly inhibit the growth of colon adenocarcinoma cells [44]. Lei et al explored a potential anti-colorectal carcinoma effect of sophoridine using mice with tumor xenograft of SW480 cells subcutaneously in the mice armpit [45]. The anti-tumor activity of SRI (15 and 25 mg/kg, 5 days a week) was compared with 5-fluorouracil (30 mg/kg, 3 days a week), both given through i.p. injection. The experiment in SW480 tumor-bearing mice demonstrated that the higher dose of SRI had a significant effect on tumor inhibition [45]: the average tumor volume in SRI (25 mg/kg) group was significantly lower than in the control group (0.579 ± 0.144 vs 1.149 ± 0.142 cm^3^, respectively). Based on the in vitro and in vivo experiments, authors concluded that SRI acts as a pro-apoptotic factor in SW480 cells. The immunocytochemistry suggested that SRI may activate caspase-3-dependent pathways, leading to cell apoptosis. Of note, after treatment with SRI there was no change in animal behavior; the animals ate and defecated normally and their weight increased. The mice treated with 5-fluorouracil (5-FU) ate less throughout the study; they also weighed much less and developed bloody diarrhea in the later stages of the treatment [45].
- *Spica Prunellae*, the fruit spikes of the perennial plant *Prunella vulgaris* L, are used in TCM to treat poor vision, blood stasis, and edema. They are also believed to possess anti-cancer properties. Lin et al reported that the extract of *Spica Prunellae* promotes apoptosis of human CRC cells and displays anti-angiogenic activity in vitro [46]. In another study the authors investigated the anti-carcinogenic activity of *Spica Prunellae* using a mouse HT-29 cell xenograft model [46]. In the course of the experiment, the animals received the ethanol extract of SP (EESP) at the dose of 6 g/kg or saline (both p.o.) once daily, 5 days a week for 16 days [46]. Body weight and tumor growth were measured every 2 days. EESP treatment significantly reduced both, tumor volume and weight compared with control mice. Moreover, EESP treatment did not affect body weight. These results indicate that EESP effectively suppresses CRC growth in vivo without apparent signs of toxicity. The gathered data suggested that EESP promotes CRC cell apoptosis, as well as inhibits cell proliferation and tumor angiogenesis. According to Lin et al the mechanism of EESPs activity is associated with suppression of the STAT3 pathway activation and regulation of the expression of Bcl-2, Bax, Cyclin D1, CDK4, VEGF-A, and VEGFR-2 in xenograft mice [46].

### Chinese traditional medicine and CRC (human studies)

- To date, some of the most promising TCM compounds have been tested in patients with diagnosed CRC (Table 2). Recently, Zhong et al published a meta-analysis of 20 trials that evaluates the efficacy of traditional Chinese herbal therapies as an adjunctive therapy for CRC [47]. All studies taken into consideration included patients with pathologically or cytologically confirmed CRC treated with chemotherapy. Only randomized controlled trials were eligible and studies comparing chemotherapy with or without TCM were considered. Total survival rate and tumor response were regarded as the final outcome. It was found that the chemotherapy combined with TCM significantly increased 1- and 3-year survival rate (OR 2.41, 95 % confidence interval (CI) 1.32–4.41; OR 2.40, 95 % CI 1.49–3.87; 5 studies, *n* = 396) when compared with chemotherapy alone [47]. Combined therapy slowed CRC progression (OR 0.5, 95 % CI 0.32–0.77; 7 studies *n* = 269) and improved quality of life (OR 3.43, 95 % CI 2.35–5.02, 9 studies, *n* = 649) [47]. Moreover, TCM as an additive therapy significantly reduced adverse effects of chemotherapy. This meta-analysis suggests that TCM may become a potent and safe supplement to chemotherapy in the future. However, a firm conclusion could not be made because of insufficient quality of some trials included in the study [47].
- Yang et al evaluated the effectiveness of a comprehensive TCM therapy on reducing the relapse and metastasis of stage II and III CRC vs conventional Western medicine (WM) treatment [48]. There were 222 patients included into the trial with CRC on stage II or II diagnose; staging standard was referred to the guidelines of the International Union Against Cancer (IUAC). Patients were combined into 2 groups: treated with conventional WM (meant as R0 radical operation or chemotherapy or/and radiotherapy according to National comprehensive cancer network (NCCN) clinical guidelines) and treated with WM combined with TCM, such as: Sijunzi Decoction, Chaihu Shugan Powder, Bazhen Decoction, Huachansu Tablet, Quxie capsule, Pingxiao capsule [48]. The results showed that the relapse/metastasis rates in 1-, 2-, 3-, 4-, 5-year were lower in a group with TCM as adjunct therapy than in the group with chemotherapy alone. Median relapse/metastasis time was 26.5 months in the combined group and 16.0 months in group treated with western medicine only [48].
- For hundreds of years, a TCM herbal preparation called PHY906 has been used in Asia for treatment of GI disorders. PHY906 is composed of 4 main herbs: *Scutulleria baicalensis* Georgi, *Paeonia lactiflora* Pall, *Glycyrrhiza uralensis* Fisch, and *Ziziphus jujube* Mill. In vivo studies on animal CRC models showed encouraging results, demonstrating that therapy with PHY906 combined with conventional drugs is more potent and less toxic than the use of conventional drugs alone [49]. Recently, Kummar et al conducted a phase I multicenter, double-blinded, randomized, placebo controlled, dose escalation, cross-over study of PHY906 as a modulator of irinotecan chemotherapy in patients with colorectal cancer [49], with 17 cases enrolled to the phase I study. Patients were treated according to the IFL regimen (weekly dose of irinotecan 125 mg/m^2^, followed by leucovorin at 20 mg/m^2^ and 5-fluorouracil at a dose of 500 mg/m^2^) with PHY906 or placebo. The study showed a reduction in incidence of diarrhea in patients treated with PHY906, which resulted in a lower use of antidiarrheals. There was also a trend toward lower frequency and severity of vomiting in patients receiving PHY906 as opposed to placebo [49].
- Yang et al evaluated the potency of Quxie Capsule on the median survival time and quality of life in patients with advanced CRC [50]. Forty patients with CRC were enrolled to this randomized controlled clinical trial. All patients were treated by routine CRC therapy, including chemotherapy and radiotherapy, additionally 1 group was administrated with Quxie capsule. After 3 months of the treatment the fatality rate, survival time, median survival time, time to progression and quality of life were evaluated. Results showed that Quxie capsule can prolong the fatality rate, survival time, median survival time and improve quality of life in patients with CRC [50].
- In another study Luo et al evaluated the effect of Quxie Capsule and Fuzheng Capsule in reducing relapse and metastasis of CRC in stage II and II after radical operation treatment (with addition radiotherapy or/ and chemotherapy) [51]. A prospective cohort controlled study was conducted in 101 patients with CRC, from which 53 were treated with Quxie Capsule and Fuzheng Capsule and 48 were given no treatment. The rate and time of relapse and metastasis were observed after 1, 2, and 3 years. Both parameters were higher in patients treated with TCM (average time of relapse and metastasis: 26.2 ± 4.5 months in group treated with TCM and 14.2 ± 4.2 months in control group) [51]. Unfortunately, this study did not compare Quxie Capsule and Fuzheng Capsule to any form of conventional anti-CRC treatment what substantially reduces the scientific importance of the results obtained.

## Concluding remarks

Conventional treatment of GI diseases is currently limited to pure overcoming of their symptoms and often associated with severe adverse effects of the drugs used. Application of TCM is getting more and more popular among IBD and CRC patients during recent years. Careful analysis of the experimental data leads to the conclusion that TCM methods are promising and in some cases they may worthily replace conventional methods. However, there is yet insufficient number of controlled trials evaluating potential efficacy of TCM approaches in IBD and CRC. Although several products have been tested, the scientific evidence regarding their efficacy or safety has not been adequate, and the majority of studies have produced inconsistent results. There is an unquestionable need for further studies on TCM methods in the treatment of GI disorders to establish their quality and safety.
